# Cervical balance and clinical outcomes in cervical spondylotic myelopathy treated by three-level anterior cervical discectomy and fusion and hybrid cervical surgery: A CONSORT-compliant study with minimum follow-up period of 5 years

**DOI:** 10.1097/MD.0000000000025824

**Published:** 2021-05-07

**Authors:** Fanqi Meng, Shuai Xu, Yan Liang, Zhenqi Zhu, Kaifeng Wang, Haiying Liu

**Affiliations:** Department of Spinal Surgery, Peking University People's Hospital, Peking University, Beijing, P.R. China.

**Keywords:** anterior cervical discectomy and fusion, cervical alignment, cervical balance, cervical spondylotic myelopathy, hybrid cervical surgery

## Abstract

As the technology of combining with fusion and nonfusion procedure, cervical hybrid surgery (HS) is an efficacious alternative for treatment with cervical spondylotic myelopathy. While studies on cervical alignment between 3-level HS and anterior cervical discectomy and fusion (ACDF) were seldom reported. The effects of cervical imbalance on its related clinical outcomes are yet undetermined as well.

Patients with cervical spondylotic myelopathy, who underwent 3-level ACDF or HS, were included to compare cervical alignment parameters after surgery and then explore the relationship between cervical balance and clinical outcomes.

Forty-one patients with HS (HS group) and 32 patients who with ACDF (ACDF group) were reviewed from February 2007 to September 2013 with the mean follow-up of 90.3 ± 25.5 (m) and 86.3 ± 28.9 (m), respectively. Cervical alignments parameters including the C2 to C7 cervical lordosis (CL), C2 to C7 sagittal vertical axis, T1 slope. and T1SCL (T1 slope minus CL), and the clinical outcomes like neck disability index (NDI) and Japanese Orthopedic Association (JOA) score were measured and recorded preoperatively (PreOP), intraoperatively, and on the first preoperative day and the last follow-up (FFU). The balance and imbalance groupings were sorted based on the T1SCL: T1SCL≤20°,balance; T1SCL > 20°, imbalance.

We found significant improvements (*P* < .001) in NDI and JOA at intraoperatively and FFU after ACDF and HS, and no difference on cervical alignment and clinical outcomes between the 2 procedures on the basis of intergroup comparisons. By between-subgroups comparisons, however, we found significant differences in CL and T1SCL at PreOP (*P* < .05). Nonetheless, there was no significant difference on the clinical outcomes between balance and imbalance subgroups at FFU at PreOP (*P* > .05), indicating that the change of T1SCL was not correlated to NDI and JOA at FFU.

Both HS and ACDF groups showed significant clinical improvements after surgery. There was no correlation between cervical balance and clinical symptoms.

## 1 Introduction

Research on the spino-pelvic alignment in sagittal plane has been widely reported. And the spino-pelvic mis-alignment can result in global sagittal imbalance in the upright standing position.^[1,2]^ One significant predictor of disability is the mismatch (≥ 9°) between lumbar lordosis (LL) and pelvic incidence (PI).^[3,4]^ Recently, T1 slope minus C2-C7 cervical lordosis (T1SCL), analogous to PI-LL, has been applied to cervical alignment parameters,^[5]^ when T1SCL ≤20°, cervical spine is considered balanced, and when T1SCL> 20°, cervical spine is regarded as imbalanced. However, this indicator was rarely reported in the spine imbalance studies.

Anterior cervical discectomy and fusion (ACDF) is a standard and accepted procedure for treating cervical spondylotic myelopathy (CSM). Since recent decades, cervical total disc replacement has been designed to preserve the motion of the operated levels. As the concept of combining with fusion and arthroplasty technology where appropriate, cervical hybrid surgery (HS) was then proposed. Contrasted with posterior approach, both HS and ACDF showed superiority in the treatment of CSM with less invasion, preservation of posterior muscle-ligament complex and direct decompression.^[6,7]^ Though postoperative cervical sagittal alignment and cervical-balance were well established after ACDF,^[7]^ the HS, a technique with or without fusion, was different from ACDF in the range of motion (ROM)-preservation of index segments and less loading on adjacent segments. The study on cervical alignment between 3-level HS and ACDF, with larger iatrogenic interference on cervical spine, remains seldom reported. The multilevel procedure was chosen as it was different from single- or double-level surgery in terms of operation time, exposure zone, tissue stretch, and biomechanics.

The primary goal of surgery is to improve the quality of life and neurological function regardless of the cervical balance status. While studies on lumbar surgery found no correlation between pelvic balance with clinical outcomes,^[8–10]^ the role of cervical balance has not been determined yet. Therefore, this study is performed on the patients with CSM underwent consecutive t3-level HS or ACDF more than 5 years, to compare long-term cervical alignment parameters and clinical outcomes, and then to explore the relationship between cervical balance status and clinical outcomes.

## 2 Methods

### 2.1 Participants and surgical procedure

The study included patients with CSM who underwent HS or ACDF from February 2007 to September 2014. And it was approved by the local institutional review board and all the patients have signed informed consent. The inclusion criteria and exclusion criteria were shown in Table 1.

**Table 1:** The inclusion criteria and exclusion criteria in this study.

Inclusion criteria
Patients required surgery after at least 6-mo uncontrolled conservation treatment.
Patients were performed consecutive 3-level HS or ACDF.
Patients possessed intact radiographic and clinical outcomes.
Exclusion criteria
Patients’ radiological alignment parameters were too unclear to measure (n = 8).
Patients were underwent previous cervical surgery (n = 2).
Patients had operation for cervical spine fracture or infection (n = 1).
Patients were with follow-up less than 5 years or with incomplete information (n = 13).
Patients were in loss to follow-up for unwilling cooperation or mortality (n = 6).

ACDF = anterior cervical discectomy and fusion, HS = hybrid surgery.

The main indications for multilevel anterior-approach were symptomatic multilevel degenerative disc disease with neuro-dysfunction after a 6-month conservative treatment.^[11]^ Generally, fusion technique was applied to more severe degenerative segment while nonfusion was used to the degenerative segment with ROM ≥6°; the loss of intervertebral space was < 80% of the normal adjacent segment; no instability of the segment; no severe loss of lordosis; no obvious canal stenosis; and no obvious osteoporosis, although there was no consensus of the threshold for third to six.^[12]^

One senior spine surgeon performed all the cases of HS or ACDF in this study: During surgery, patients were placed in a supine position. An anterior right-sided incision was made and standard Smith-Robinson approach to the cervical spine was performed. Bilateral discectomy and uncinated process resection were conducted even with unilateral symptoms. After complete decompression, the artificial disc together with poly-ether-ether-ketone (PEEK) cage was implanted in HS, while 3 PEEK cages were inserted in ACDF. All patients were instructed to wear collar for 2 months after surgery.

The type of artificial disc was Prodisc-C (Depuy Synthes, New Brunswick, USA) and the PEEK cage was MC+ (LDR Medical, Troyes, France). In HS group, there were 36 cases implanted 2 cages+1 artificial disc and 5 cases with 1 cage+2 artificial discs; In ACDF group, 13 of 32 patients were implanted with anterior rigid plate.

### 2.2 Cervical alignment parameters

**Figure 1:**
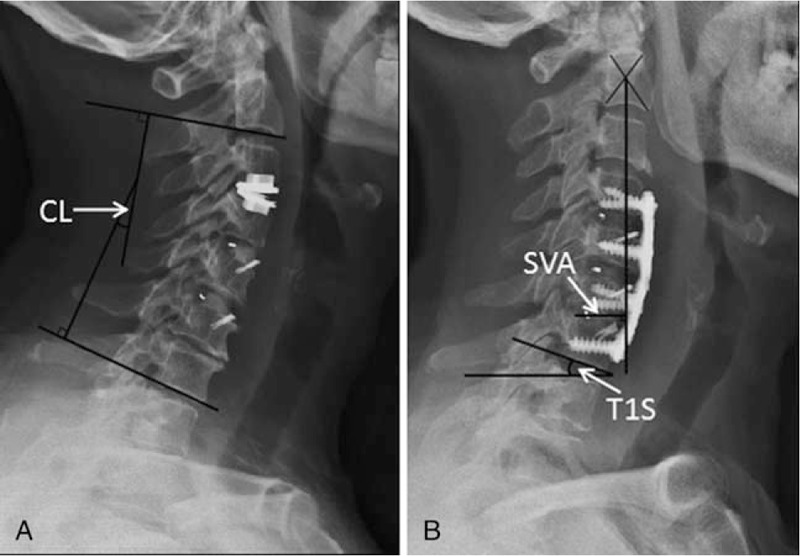
Measurements of cervical alignment parameters on standard lateral X-ray. (A) Measurement of CL; (B) measurement of SVA and T1S. CL = C2-C7 cervical lordosis, SVA = C2-C7 sagittal vertical axis, T1S = T1 slope; CL was the angle from lower endplate of C2 to lower endplate of C7, SVA was measured from C2 plumb line to posterior margin of the upper endplate of C7; T1S was from upper endplate of T1 to horizontal line.

The parameters were measured from standing lateral X-ray of cervical spine (Fig. 1). The following cervical alignment parameters were included: C2 to C7 cervical lordosis (CL), C2 to C7 sagittal vertical axis (SVA), T1S and T1SCL. CL was the angle from lower endplate of C2 to lower endplate of C7; SVA was measured from C2 plumb line to posterior margin of the upper endplate of C7; T1S was from upper endplate of T1 to horizontal line. T1SCL was used to evaluate the cervical sagittal balance (T1SCL≤20°, balance; T1SCL> 20°, imbalance).^[13]^ The radiograph was obtained at preoperation (PreOP), immediately after operation (ImOP), and final follow-up (FFU).

CL, SVA, and T1S were measured by 3 experienced surgeons with at least duplicated measurement. Intraobserver and interobserver reliability were assessed using intraclass correlation (ICC) coefficients (excellent (>0.90), good (0.71–0.90), fair (0.51–0.70) and poor (<0.51)).

### 2.3 Clinical outcomes assessment

Life quality was assessed by neck disability index (NDI) and Japanese Orthopedic Association (JOA) score, which were evaluated at PreOp, ImOP, and FFU by questionnaire. The recovery rate (RR) of JOA was calculated by the Hirabayashi method: RR (%)= (PostOP JOA−PreOP JOA)/(17−PreOP JOA) × 100.

### 2.4 Balance and imbalance subgroups

Further analysis was conducted to explore whether clinical outcomes differed between:

1. Patients with an imbalanced cervical spine at PreOP (subgroup U) who regained cervical balance at FFU (subgroup UB) compared with those who did not regain cervical balance (subgroup UU).
2. Patients with a balanced cervical spine at PreOP (subgroup B) that was maintained at FFU (subgroup BB) compared with those who lost cervical balance at FFU (subgroup BU).
3. Patients with cervical balance at FFU (subgroup UB + subgroup BB) either maintained at PreOP (subgroup BB) or regained by surgery (subgroup UB) compared with those with an imbalanced cervical at FFU (subgroup UU+ subgroup BU) whatever the cervical-balance status (subgroup B or subgroup U) at PreOP.

Moreover, whether the change in T1SCL correlated with clinical outcomes needed to be confirmed. It contained potential correlations between the degree of the change in T1SCL and NDI at FFU, change of NDI at FFU, JOA scores at FFU, and RR of JOA scores at FFU.

### 2.5 Statistical analysis

Student *t* test or Mann–Whitney *U* test was used to compare cervical alignment parameters and clinical outcomes between HS and ACDF groups, and between balanced and imbalanced subgroups with the same surgery. Student *t* test was also used to compare clinical outcomes between balanced and imbalanced subgroups at FFU. ANOVA analysis was used to compare measurements among PreOP, ImOP, and FFU in the same group. *χ*^2^ test or Fisher test was performed on dichotomous between 2 groups. Pearson correlation analysis was utilized between T1SCL and clinical outcomes. The statistical analysis was performed using IBM SPSS Statistics 22.0 (International Business Machines Corporation, Armonk, NY) and *P* < .05 was defined as statistical significance.

## 3 Results

A total of 103 cases were originally included and 11 of them were excluded at baseline. Finally, 73 participants completed 5-year follow-up with a completing rate of 79.3%. Of whom, 41 patients underwent HS (HS group) and 32 patients were treated with ACDF (ACDF group). There was no significance in terms of age, gender, and body mass index between the 2 groups. The mean follow-up time was 90.3 ± 25.5 (m) and 86.3 ± 28.9 (m) in HS group and ACDF group, respectively (*P*> .05). There was no difference on distribution of operated segments between the 2 groups (*P* = .626), so were operation time (*P* = .083) and blood loss (*P* = .061) (Table 2). ICC for intraobserver reliability was 0.909 on CL, 0.943 on SVA, and 0.935 on T1S; ICC for interobserver reliability was 0.872 on CL, 0.914 on SVA and 0.844 on T1S.

**Table 2:** Demographic characteristics and surgery information between HS and ACDF group.

	HS group	ACDF group	*P*
Gender (M/F)	20/21	17/15	.713
Age (yr)	55.5 ± 8.0	57.2 ± 8.3	.494
BMI (kg/m^2^)	25.4 ± 3.1	24.8 ± 3.4	.429
DM	8	3	.325
Smoking	11	6	.418
Follow-up (mo)	90.3 ± 25.5	86.3 ± 28.9	.523
Operated segments			.626
C3-C6	11	7	
C4-C7	30	25	
Operation time (min)	109.1 ± 17.8	101.1 ± 21.1	.083
Blood loss (mL)	99.4 ± 83.3	71.3 ± 37.1	.061

ACDF = anterior cervical discectomy and fusion, BMI = body mass index, DM = diabetes mellitus, F = female, HS = hybrid surgery, M = male.

### 3.1 Comparisons between ACDF and HS

In terms of cervical alignment, CL was of no significance at ImOP contrasted with that of PreOP, but an improvement at FFU in both groups (*P* = .025 and *P* = .043) and HS procedure had slightly better efficacy in alignment restoring (*P* = .046). There was no significance on SVA and T1SCL at FFU compared with PreOP and between the 2 groups at ImOP and FFU. A slightly larger T1S at ImOP and FFU were measured in HS group but not in ACDF group. Clinical outcomes showed there were all significant improvements (all *P* < .001) in NDI and JOA at ImOP and FFU. Patients in HS group gained a higher improvement in NDI but not in RR JOA (Table 3).

**Table 3:** Comparison on cervical alignment and clinical outcomes between HS and ACDF groups.

	HS group	ACDF group	*P*
Outcomes at PreOP			
CL (°)	11.8 ± 11.4	6.8 ± 14.3	.105
SVA (cm)	1.5 ± 1.0	2.0 ± 1.0	.034
T1S (°)	23.2 ± 8.3	24.1 ± 8.4	.659
T1SCL (°)	11.4 ± 9.5	17.3 ± 9.9	.011
NDI	39.1 ± 3.8	38.0 ± 3.0	.153
JOA	11.2 ± 1.7	10.3 ± 1.9	.041
Outcomes at ImOP			
CL (°)	14.9 ± 9.1	11.1 ± 10.2	.095
SVA (cm)	2.2 ± 1.0^†^	2.6 ± 1.3	.191
T1S (°)	26.9 ± 7.7^∗^	26.1 ± 8.0	.698
T1SCL (°)	11.9 ± 9.4	15.1 ± 8.9	.151
NDI	19.9 ± 4.4^†^	19.3 ± 6.9^†^	.648
Δ_1_NDI^||^	19.8 ± 4.8	19.0 ± 6.9	.614
JOA	14.9 ± 0.8^†^	14.6 ± 1.4^†^	.268
RR_1_ JOA(%)^||^	62.7 ± 13.7	63.8 ± 23.5	.806
Outcomes at FFU			
CL (°)	16.7 ± 7.9^‡^	12.6 ± 9.0^‡^	.046
SVA (cm)	1.7 ± 1.2	1.9 ± 1.2	.494
T1S (°)	26.7 ± 7.0^‡^	26.0 ± 7.3	.666
T1SCL (°)	10.0 ± 7.9	13.3 ± 9.9	.118
NDI	9.9 ± 3.2^§^	12.5 ± 8.2^§^	.123
Δ_2_NDI^||^	29.8 ± 4.6	25.8 ± 8.2	.013
JOA	16.2 ± 1.0^§^	15.7 ± 1.9^§^	.281
RR_2_ JOA(%)^||^	85.4 ± 18.8	81.8 ± 29.4	.547

ACDF = anterior cervical discectomy and fusion, CL = C2-C7 cervical lordosis, FFU = final follow-up after operation, HS = hybrid surgery, ImOP = immediately after operation, JOA = Japanese Orthopedic Association score, NDI = neck disability index, PreOP = preoperation, RR = recovery rate, SVA = C2-C7 sagittal vertical axis, T1S = T1 slope, T1SCL = T1S minus CL.

∗ Significance on parameters between PreOP and ImOP (*P* < .05).

† Significance on parameters between PreOP and ImOP (*P* < .01).

‡ Significance on parameters between PreOP and FFU (*P* < .05).

§ Significance on parameters between PreOP and FFU (*P* < .01).

|| Δ_1_NDI is the change of NDI at ImOP compared with PreOP; RR_1_ JOA is the recovery rate of JOA at ImOP compared with PreOP; Δ_2_NDI is the change of NDI at FFU compared with PreOP; RR_2_ JOA is the recovery rate of JOA at FFU compared with PreOP.

In preoperative imbalance subgroup, there were no differences on cervical alignment parameters and clinical outcomes between the 2 procedures, so were those in preoperative balance subgroup except a larger change of NDI in HS group. On intersubgroups comparisons, there were no differences on alignment and clinical outcomes in both HS and ACDF groups except CL and T1SCL at PreOP. Although significance at PreOP, there were no differences on CL and T1SCL at FFU between the 2 subgroups (all *P*> .05) (Table 4).

**Table 4:** Comparison on cervical alignment and clinical outcomes inner- and inter balance subgroup and imbalance subgroup.

	Balance subgroup at PreOP			Imbalance subgroup at PreOP		
	HS group	ACDF group	*P*	HS group	ACDF group	*P*
Cervical alignment						
CL at PreOP (°)	14.3 ± 10.1^∗^	13.8 ± 11.5^∗^	.861	−2.6 ± 7.4	−5.0 ± 10.3	.623
CL at FFU (°)	17.2 ± 7.5	13.7 ± 9.3	.119	12.6 ± 9.9	10.8 ± 8.6	.695
SVA at PreOP (cm)	1.4 ± 1.0	1.8 ± 1.0	.152	2.2 ± 1.0	2.4 ± 0.9	.653
SVA at FFU (cm)	1.7 ± 1.0	1.8 ± 1.1	.657	1.9 ± 2.2	2.1 ± 1.5	.874
T1S at PreOP (°)	23.2 ± 8.6	24.8 ± 8.5	.509	23.3 ± 7.3	22.9 ± 8.5	.931
T1S at FFU (°)	26.8 ± 7.3	25.1 ± 6.9	.396	25.8 ± 4.4	27.8 ± 8.1	.664
T1SCL at PreOP (°)	8.9 ± 7.9^∗^	11.0 ± 6.1^∗^	.305	25.8 ± 3.8	27.8 ± 4.1	.328
T1SCL at FFU (°)	9.5 ± 7.0	11.4 ± 10.0	.454	13.2 ± 12.4	16.5 ± 9.3	.524
Clinical outcomes						
NDI at PreOP	39.7 ± 3.8	38.1 ± 3.0	.104	39.4 ± 4.4	37.9 ± 3.0	.380
NDI at FFU	9.7 ± 3.3	12.8 ± 8.9	.176	11.0 ± 3.1	12.0 ± 7.3	.758
ΔNDI^†^	30.0 ± 4.7	25.5 ± 9.1	.025	28.5 ± 3.9	26.2 ± 6.8	.465
JOA at PreOP	11.1 ± 1.8	10.3 ± 1.7	.140	12.0 ± 0.6	10.3 ± 2.1	.085
JOA at FFU	16.3 ± 1.0	15.6 ± 2.2	.230	15.7 ± 1.0	16.0 ± 1.4	.625
RR JOA (%)^†^	87.4 ± 18.2	78.7 ± 34.6	.251	74.4 ± 20.0	87.0 ± 17.5	.208

ACDF = anterior cervical discectomy and fusion, CL = C2-C7 cervical lordosis, FFU = final follow-up after operation, HS = hybrid surgery, JOA = Japanese Orthopedic Association score, NDI = neck disability index, PreOP = preoperation, RR = recovery rate, SVA = C2-C7 sagittal vertical axis, T1S = T1 slope, T1SCL = T1S minus CL.

∗ Significance on parameters between balance and imbalance subgroups with the same surgery (*P* < .01).

† ΔNDI is the change of NDI at FFU compared with PreOP; RR JOA is the recovery rate of JOA at FFU compared with PreOP.

There were 35 balanced cases and 6 imbalanced cases in HS group at PreOP while 38 balanced cases and 3 imbalanced cases at FFU. Similarly in ACDF group, 20 cases were in balance subgroup and 12 cases were in imbalance subgroup at PreOP. After ACDF, 4 cases indulged in imbalance from balance while 3 patients regained balance from imbalance (Table 5). There were more cases of balance in HS group than ACDF group at PreOP (*P* = .025), but the capacity of balance maintaining (*P* = .175) and imbalance correction (*P* = .688) between the 2 procedures was comparable.

**Table 5:** Migration of patients to balanced and unbalanced cervical subgroups after HS and ACDF.

	Balance subgroup at FFU	Imbalance subgroup at FFU
HS group		
Balance subgroup at PreOP		
35 subgroup B	33 subgroup BB^∗^	2 subgroup BU^∗^
Imbalance subgroup at PreOP		
6 subgroup U	5 subgroup UB^∗^	1 subgroup UU^∗^
Total		
41 B+U	38 BB+UB	3 BU+UU
ACDF group		
Balance subgroup at PreOP		
20 subgroup B	16 subgroup BB	4 subgroup BU
Imbalance subgroup at PreOP		
12 subgroup U	9 subgroup UB	3 subgroup UU
Total		
32 B+U	25 BB+UB	7 BU+UU

ACDF = anterior cervical discectomy and fusion, B = balance, FFU = final follow-up after operation, HS = hybrid surgery, PreOP = preoperation, U = unbalance.

∗ BB means patients in cervical balance at PreOP and also in cervical balance at FFU; BU means patients in cervical balance at PreOP but in cervical imbalance at FFU; UB means patients in cervical imbalance at PreOP but in cervical balance at FFU; UU means patients in cervical imbalance at PreOP and still in cervical imbalance at FFU.

### 3.2 Correlation between cervical balance and clinical outcomes

Integrating data of HS and ACDF groups, analysis on balance status and clinical outcomes were shown in Table 6. Patients in subgroup U at PreOP belonging to subgroup UB compared with subgroup UU showed no differences in NDI or JOA scores and their changes (all *P*> .05), the same with the comparisons between subgroups BB and BU. There was also no significance in patients with cervical balance at FFU (subgroup UB+subgroup BB) compared with imbalanced cervical at FFU (subgroup UU+subgroup BU). Furthermore, the correlation analysis between the change of T1SCL and clinical outcomes showed no relationship between the change of T1SCL at FFU and NDI at FFU, change of NDI at FFU, JOA scores at FFU, and RR of JOA scores at FFU (all *P*> .05) (Table 7).

**Table 6:** Clinical outcomes between balance and imbalance subgroups at FFU.

	Balance at PreOP			Imbalance at PreOP			Balance/imbalance at PreOP		
	Balance at FFU	Imbalance at FFU	*P*	Balance at FFU	Imbalance at FFU	*P*	Balance at FFU	Imbalance at FFU	*P*
NDI at FFU	12.3 ± 6.5	9.5 ± 3.9	.430	10.9 ± 6.2	9.5 ± 3.7	.591	11.2 ± 6.2	9.5 ± 3.6	.403
ΔNDI^∗^	27.3 ± 6.5	26.3 ± 3.8	.760	28.3 ± 7.0	30.2 ± 5.5	.527	28.1 ± 6.8	28.6 ± 5.1	.816
JOA at FFU	15.7 ± 1.3	16.5 ± 1.0	.265	16.0 ± 1.6	16.2 ± 1.3	.831	15.9 ± 1.5	16.3 ± 1.2	.484
RR JOA (%)^∗^	79.7 ± 18.7	90.0 ± 20.0	.363	84.1 ± 25.6	86.7 ± 21.6	.817	83.2 ± 24.2	88.0 ± 19.9	.554

FFU = final follow-up after operation, JOA = Japanese Orthopedic Association score, NDI = neck disability index, PreOP = preoperation, RR = recovery rate.

∗ ΔNDI is the change of NDI at FFU compared with PreOP; RR JOA is the recovery rate of JOA at FFU compared with PreO.

**Table 7:** Correlation analysis between the change of T1SCL and clinical outcomes.

	r	*P*
ΔT1SCL^∗^ and NDI at FFU	0.071	.571
ΔT1SCL and ΔNDI^∗^	−0.049	.699
ΔT1SCL and JOA at FFU	−0.064	.611
ΔT1SCL and RR JOA^∗^	−0.077	.540

FFU = final follow-up after operation, JOA = Japanese Orthopedic Association score, NDI = neck disability index, r = correlation coefficient, RR = recovery rate, T1SCL = T1 slope minus C2-C7 cervical lordosis.

∗ ΔT1SCL is the change of T1SCL at FFU; ΔNDI is the change of NDI at FFU; RR JOA is the recovery rate of JOA at FFU.

## 4 Discussion

It was reported that the maintenance and improvements of cervical sagittal balance might have an effect on clinical efficacy.^[14,15]^ Which, however, was not a consensus in various procedures. The concept of cervical balance was evaluated by many factors. In most publications, cervical imbalance was defined as T1S > 40° or SVA > 40 mm.^[16]^ Recently, T1SCL was considered a landmark for evaluating cervical balance with a threshold of 20°.

Protopsaltis et al^[17]^ considered the balance between T1SCL mirrors the relationship between PI minus LL of ± 9°. Hyun et al^[5]^ found T1SCL mismatch might be associated with a greater degree of cervical spine malalignment and disability. Several studies have addressed changes in cervical alignment, focusing on sagittal balance before and after cervical surgeries.^[18,19]^ This study first performed an analysis on cervical spine balance after consecutive 3-level ACDF or HS, concluding an identified improvement on cervical balance maintaining or correction with both approaches.

In the study of spino-pelvic parameters, Maciejczak et al^[20]^ have defined balanced pelvis and unbalanced pelvis, they proposed that disturbances in pelvic balance may affect the quality of life. But they addressed radiological improvement of pelvic balance did not correlate with clinical outcomes,^[8]^ similar to what we found. For patients with cervical imbalance, when a cervical spine had a higher T1S and worse alignment, it becomes bent over horizontally under a kyphotic force.^[21]^ Therefore, the aim for imbalanced cases was to decrease thoracic inlet and restore CL. In this study, HS or ACDF has corrected CL but not for T1S and T1SCL, which might be due to that most T1S at PreOP was in normal range (75.3%). Therefore, the study first proved that most patients who underwent such procedures were actually in cervical balance.

The difference in number of cervical-balanced patients between HS and ACDF group might be due to different surgical indication of HS and ACDF. HS was inclined to be selected for cervical-balanced cases, less degenerated adjacent segment disc and younger cohort^[7]^ but the age has no significance in our study. Multilevel surgery, with a unique superiority on reconstruction compared with single-level surgery, allows more direction decompression and adequate correction of the entity causing pressure.^[22]^ Significantly and comparably, the cervical-balance reconstruction improved after both procedures, which was due to the less incision and protection of posterior muscle-ligament complex. Sakai et al^[23]^ found postoperative cervical alignment and balance were maintained after ACDF but deteriorated following laminoplasty by a review on prospective cohort studies.^[24,25]^ In addition, cervical alignment was reconstructed by anterior tissue release, preparation of endplate bed, the shapes and sizes of implants.^[21]^

There were no intersubgroup differences on cervical alignment parameters between HS and ACDF. It was the similar approach and anatomy structure of the 2 procedures that mattered. Although the dynamic implant in HS group, preservation of ROM, and different biomechanics of 1 or 2 levels compared with ACDF seemed to put not much impact on cervical alignment, which suggested that the key role was technique itself (such as adequate tissue release and osteophyte removal) rather than the types of implants.^[23,26]^ The indifferent comparisons indicated that 3-level HS or ACDF achieved comparable efficacy on balance-maintaining and reconstruction.

The comparable clinical outcomes proved an identified efficacy for CSM patients who went through HS or ACDF. On earth, the goal of surgical procedures was to decompress the spinal cord and improve neurological function.^[27]^ The reasons for slightly superiority of change of NDI in the HS group might be as follows. First, artificial disc made an impact on ROM-preservation contrasted with ACDF, which avoided exceeding ROM of adjacent segments. In addition, JOA was the patient-reported assessment tool to address each of these domains,^[28]^ while NDI was a mix of functional- and pain-status inquiries.^[29,30]^

An analysis was finally performed by combining 2 groups with little heterogeneity. There were no differences on clinical outcomes between balance and imbalance subgroups at FFU, regardless of balance or imbalance at PreOP. It was possible that decompression and stabilization, rather than balance status, were sufficient to obtain improvement in most sections.^[8,9]^ Although the significance of disorders on spino-pelvic alignment in global sagittal imbalance of the whole body,^[1,2]^ the cervical alignments seemed probable not as important as lumbar spino-pelvic parameters. The adequate decompression spared compensated space for the spinal cord recruitment even if the volume of spinal canal could be affected by alignment.^[14,31]^ Finally, the global spine and lower extremities balance might be a result of compensatory mechanisms aiming at adapting body posture in response to regional cervical-imbalance.^[32,33]^

There were some limitations of this study. First, the sample of both groups was little and thus there might be some reporting bias. A larger population could support strength verification with a cohort study. In addition, there was no subgroup analysis on whether rigid-plating was used in ACDF, although anterior rigid-plating may have an impact on cervical balance. Finally, the conclusion was only suitable for the patients with CSM who underwent 3-level HS or ACDF; it is not suitable for other cervical diseases or procedures.

## 5 Conclusions

Based on this retrospective study (evidence level III), there were identified improvements on cervical balance maintaining or imbalance-correction after both HS and ACDF, and the balance status was comparable between the 2 groups. There was no significance in cervical alignment and clinical outcomes between the 2 procedures either at PreOP or at FFU. Most patients were in cervical sagittal balance and few balance status of CSM cases needed to be paid extra attention to. No correlation between the cervical balance and clinical outcomes was found, neither was between T1SCL change and clinical outcome improvement, no matter what the cervical-balance status was at PreOP (recommendation Grade C).

## Acknowledgment

The authors acknowledge Houshan Lv who contributed toward the study by making substantial contributions to the design and the acquisition of data.

## Author contributions

**Conceptualization:** Haiying Liu, Fanqi Meng.

**Data curation:** Fanqi Meng, Zhenqi Zhu, Haiying Liu.

**Formal analysis:** Fanqi Meng, Shuai Xu.

**Investigation:** Zhenqi Zhu, Kaifeng Wang.

**Methodology:** Fanqi Meng; Shuai Xu, Yan Liang.

**Project administration:** Yan Liang, Haiying Liu.

**Resources:** Fanqi Meng; Yan Liang, Junhao Deng.

**Software:** Fanqi Meng, Shuai Xu, Junhao Deng.

**Validation:** Shuai Xu; Visualization: Haiying Liu.

**Visualization:** Haiying Liu.

**Writing – original draft:** Fanqi Meng, Shuai Xu.

**Writing – review & editing:** Fanqi Meng, Shuai Xu, Haiying Liu.
